# Chidamide, a subtype-selective histone deacetylase inhibitor, enhances Bortezomib effects in multiple myeloma therapy

**DOI:** 10.7150/jca.61602

**Published:** 2021-08-27

**Authors:** Yanjuan He, Duanfeng Jiang, Kaixuan Zhang, Yinghong Zhu, Jingyu Zhang, Xuan Wu, Jiliang Xia, Yan Zhu, Lang Zou, Jian Hu, Yajuan Cui, Wen Zhou, Fangping Chen

**Affiliations:** 1 Department of Hematology, Xiangya Hospital, Central South University, Changsha, Hunan, China.; 2 Department of Hematology, The 3rd Xiangya Hospital, Central South University, Changsha, China.; 3 Cancer Research Institute, School of Basic Medical Sciences, Central South University, Changsha, China.; 4 Department of Hematology, The Second Xiangya Hospital, Central South University, Changsha, China.

**Keywords:** Multiple myeloma, Chidamide, Bortezomib, Reactive oxygen species

## Abstract

Drug resistance is the major cause for disease relapse and patient death in multiple myeloma (MM). It is an urgent need to develop new therapies to overcome drug resistance in MM. Chidamide (CHI), a novel oral HDAC inhibitor targeting HDAC1, 2, 3 and 10, has shown potential therapeutic effect in MM. In this study, we determined that CHI exhibited significant anti-tumor effect on MM cells both *in vitro* and *in vivo*, which was positively correlated with the expression of HDAC1. Meanwhile, CHI enhanced Bortezomib (BTZ) effects synergistically in MM cells and a combination of CHI with BTZ induced myeloma cell apoptosis and G0/G1 arrest *in vitro* and *in vivo*. Mechanistically, the synergistic anti-tumor effect of CHI and BTZ was related with the increased production of reactive oxygen species (ROS) dependent DNA damage and the changes of cell apoptosis and cycle pathways. Our data indicate that CHI may be a suitable drug to sensitize BTZ in MM cells, which provides novel insight into the therapy for MM patients.

## Introduction

Multiple myeloma (MM) is a malignant disease characterized by proliferation of clonal plasma cells in the bone marrow and accompanied by detectable monoclonal immunoglobulins (M protein) in patients' serum or urine. Together with autologous stem cell transplantation (ASCT), multiple novel chemotherapeutic drugs such as proteasome inhibitors, immunomodulatory drugs, alkylating agents and corticoids have significantly increased response rates and patients' survival in MM in the past decades 1, 2. However, MM is still a difficult-to-cure disease due to inevitable drug resistance, and development of novel therapies are urgently required.

The two major types of epigenetic modifications - DNA methylation and histone modification, both are important in the pathogenesis of MM. Though targeting DNA methylation has not yet been fully developed, the histone deacetylases inhibitors (HDACi) have shown promising therapeutic effects in MM 3. Protein acetylation has various functions including modulation of gene expression, DNA replication and repair, cell cycle progression, cytoskeletal reorganization and protein chaperone activity 4. Increased expression of HDACs has been identified in MM cells. High expression levels of Class I HDAC, particular HDAC1, are associated with poor prognosis in MM disease 5. The HDACi as single agent induces MM cell death mainly through the inhibition of class I HDAC 6. The class I specific HDACi, romidepsin, along with the pan-HDACi dacinostat (LAQ824), belinostat, vorinostat, and panobinostat showed clearly anti-myeloma cells* in vitro* and *in vivo* 7, 8. Moreover, HDACi panobinostat, in combination with bortezomib and dexamethasone, has been approved as the first HDAC inhibitor to treat MM 9, 10.

Chidamide (CHI) (CS055/HBI-8000), discovered and developed by Chipscreen Biosciences, is a novel orally active HDACi with subtype selective activity against HDAC1, 2, 3 and 10 11. CHI was approved by the Chinese Food and Drug Administration (CFDA) for the treatment of recurrent or refractory peripheral T-cell lymphoma (PTCL) patients in December 2014. CHI was the first listed benzamide class of HDACi for global clinical trials in solid tumors including non-small cell lung cancer, breast cancer and prostate cancer in the United States and China. As an epigenetic modulator, CHI induced tumor cell growth arrest and apoptosis and enhanced cellular antitumor immunity 12-14. CHI exhibited significant cytotoxicity against MM cells co-cultured with bone mesenchymal stromal cells and CHI-pretreated osteoclasts in recent studies 15-17.

The mechanism of proteasome inhibitor Bortezomib (BTZ), leading to disruption of intracellular protein metabolism, are well characterized 18, 19. HDAC1 overexpression conferred resistance to BTZ in MM cells, and administration of the HDACi romidepsin restored sensitivity to BTZ in HDAC1-overexpressing cells both *in vitro* and *in vivo* 20. Based on preclinical data, combining HDACi with proteasome inhibitors such as BTZ represent an attractive strategy for the treatment of patients with MM 8. CHI, an improved and cheaper HDACi, has also achieved exciting result on PTCL therapy. Thus, it is meaningful to explore the single drug anti-myeloma of CHI or synergistic anti-myeloma activity of CHI with BTZ in MM.

In this study, we found that the expression level of HDAC1 was different in various MM cell lines and higher expression of HDAC1 in MM cell line resulted in more sensitivity to CHI. Meanwhile, the cell line resistant to BTZ had a higher expression level of HDAC1 and was more sensitive to CHI than wild type cell line. The effect of single drug CHI on MM cells, and the synergistic anti-myeloma effect of CHI and BTZ *in vitro* and *in vivo* was confirmed. Moreover, the potential mechanism of CHI synergized with BTZ was further explored in this study. Taken together, our findings will provide a novel and individuation therapeutic strategy for patients with MM.

## Methods

### Drugs and reagents

CHI was kindly provided by Shenzhen Chipscreen Biosciences, Ltd (Shenzhen, China). BTZ was obtained from BSP Pharmaceuticals S.p.A (Lazio, ITA), dissolved in saline solution as a stock solution for 5 mmol/L, aliquoted and stored at -80 °C. The following antibodies were used: rabbit anti-HDAC1 (catalog #AF0178), rabbit anti-cleaved Caspase-3 (catalog #AF7022), rabbit anti-cleaved PARP (catalog #AF7023), rabbit anti-cleaved Caspase-8 (cleaved-Asp384; catalog #AF5267), rabbit anti-γ-H2AX (Tyr143; catalog #AF8482), mouse monoclonal anti-β-actin (catalog #T0022) and goat anti-rabbit Alexa Fluor 594 (catalog #S0006) were purchased from Affinity Biosciences (Cincinnati, OH, USA). Rabbit anti-Ki67 (catalog #GB13030-2) was purchased from Servicebio (Woburn, MA). Matrigel (catalog #356237) was purchased from BD Biosciences Discovery Labware (Two Oak Park, Bedford, MA).

### Cell culture

The human MM cell lines XG1, KMS-11, KMS-28, ANBL-6, RPMI-8226, ARP-1 and OCI-my5 were obtained from the Cancer Research Institute of Central South University. All MM cells were grown in RPMI 1640 (Gibco, USA) medium supplemented with 10% fetal bovine serum (Gibco, USA) at 37 °C in a 5% CO_2_ incubator.

### Primary samples

Newly diagnosed or relapsed MM cases were defined according to the classification in the International Myeloma Working Group (IMWG) guidelines 21. Two cases of newly diagnosed MM and one case of relapsed MM bone marrow samples were obtained from the Xiangya Hospital, Central South University. This study was approved by the Xiangya Hospital Ethics Review Board in accordance with the Declaration of Helsinki. Acquisition of bone marrow samples was performed with the informed consents of the patients. Mononuclear cells were isolated by density gradient centrifugation using Lymphoprep™ (Solarbio, China) and cultured in RPMI 1640 supplemented with 10% fetal bovine serum, 100 U/ml penicillin, and 100 μg/ml streptomycin (1 × P/S). The clinical information of clinical samples was described in Supplementary Table S1.

### Cell viability assay

The indicated cells were planted in the 96-well plates (5 × 10^4^ cells / well) and treated with different concentrations of CHI or combined with BTZ for 48 hours. Cell viability was assessed using the CCK-8 cell proliferation kit according to the manufacturer's instructions (Dojindo Laboratories, Kumamoto, Japan).

### Quantification of the synergism of CHI with BTZ

XG1 and ARP-1 cells were treated with different doses of CHI and BTZ in monotherapy and in combinations for 48 hours. The following doses of CHI (in μmol/L) and BTZ (in nmol/L) were used: 0:0, 0.5:0.5, 1:1, 2:2, 4:4, 8:8, 16:16, 32:32. The combination index (CI) was calculated by CompuSyn for Drug Combinations and General Dose-Effect Analysis (ComboSyn, Inc. 599 MillRun, Paramus, NJ, 07653, USA), which was based on the Chou Talalay method 22 with the following interpretation: CI > 1: antagonistic effect, CI = 1: additive effect and CI < 1: synergistic effect.

### Soft agar colony forming assay

The effect of CHI and BTZ on ARP-1 cells was determined using a soft agar colony forming assay 23. Briefly, 1 × 10^6^ ARP-1 cells were treated with 1.0 μmol/L of CHI, 5.0 nmol/L of BTZ or the combination for 24 hours. Then the cells were counted and 1.5 × 10^3^ cells were plated in duplicate containing 0.5 mL of RPMI 1640 medium with 20% FBS, 0.28% agarose (low gelling temperature, Dalian Meilun Biotechnlogy Co., China) into 12-well tissue-culture plates, where pre-placed with 1.0 mL of RPMI 1640 medium with 20% FBS, 0.58% agarose at the bottom. After 8 days of incubation at 37°C in a 95% humidified atmosphere containing 5% CO_2_, colonies were counted using an inverted microscope and a graph was plotted.

### Cell cycle and cell apoptosis detection

For the cell cycle assay, indicated cells were treated with different doses of CHI or combined with BTZ for 24 hours, and 2 × 10^5^ cells were collected and fixed with 75%-80% ethanol at -20 °C for 24 hours. After centrifugation, cells were washed with phosphate buffer solution (PBS), and then incubated with propidium iodide (PI) and RNase-A (US Everbright Inc, USA) for 30 minutes at room temperature. DNA contents were analyzed by flow cytometry. For the cell apoptosis assay, cells were treated with different doses of CHI or combined with BTZ for 48 hours. Cells (2 × 10^5^) were harvested at various intervals after treatment, washed with ice-cold PBS and resuspended in 400 μL binding buffer. 20 μg/mL FITC-Annexin V and 5 μg/mL PI were added and cells were incubated for 15 minutes in a dark environment, according to the manufacturer's instructions of FITC-Annexin V and propidium iodide (PI) apoptosis kit (US Everbright Inc, USA).

### Quantitative real-time polymerase chain reaction (qRT-PCR)

Total RNA was isolated using Trizol reagent (Invitrogen, USA). Gene expression (mRNA) was analyzed using the Mastercycler ep realplex2 (Eppendorf, GER) in a two-step real-time PCR (95 °C for 5 minutes, followed by 40 cycles of 95 °C for 10 seconds and 60 °C for 30 seconds). All PCR reactions were run in triplicate, and mRNA levels of target genes relative to β-actin were calculated using the 2**^-^**^ΔΔCT^ method. The primer sequences used for PCR are listed in Supplementary Table S2.

### Western blot

The following antibodies were used: antibodies against β-actin, cleaved caspase 3, cleaved caspase 8, cleaved PARP-1, γ-H2AX, HDAC1, HDAC2 and HDAC8 (Affinity, USA). Samples were prepared and resolved on 10% SDS-PAGE, and blotted onto 0.22 μm PVDF membranes (EMD Millipore Corporation, Billerica, MA, USA). Blots were visualized using the enhanced chemiluminescence (ECL) reagents (Thermo Scientific, USA).

### Transfection of HDAC1 cDNA

ARP-1 cells treated with 1.0 μmol/L CHI or not were transfected with HDAC1 cDNA (Origene Technologies Inc., Rockville, MD, USA) using FuGENE HD Transfection Reagent according to the manufacturers' protocol. To determine the efficiency of cDNA, HDAC1 expression was assessed by Western blot assays.

### Immunofluorescence assay

ARP-1 cells were cultured with 1.0 μmol/L CHI alone or combined with 5.0 nmol/L BTZ for 24 hours. Cells were harvested and dropped in the glass coverslips, then were fixed with 4% paraformaldehyde for 20 minutes, followed by three PBS rinses, permeabilized with 0.1% Triton X-100 (Sigma) for 15 minutes and blocked with 5% BSA in PBS for 1 hour at room temperature. The samples were then stained overnight at 4 °C with primary antibody against phospho-H2AX (1: 200, Affinity, USA), followed by incubation with Alexa Fluor 594 goat anti-rabbit IgG (Affinity, USA) for 1 hour at room temperature in the dark, and then were counterstained using DAPI (Sigma, USA). Subsequently, the coverslips were mounted on the glass slides. The cells were scanned and images were captured by confocal fluorescence microscope (Olympus, Japan).

### Measurement of ROS Generation

Cells were pretreated with or without 15.0 mmol/L N-Acetyl-L-cysteine (NAC) for 2 hours at 37 °C and then incubated with various drugs for indicated times. Then the cells were washed with PBS, resuspended in RPMI 1640 medium containing 10.0 μmol/L of 2,7-dichlorodihydro-fluorescein diacetate (DCFH-DA) (Beyotime, China), and incubated at 37 °C for 20 minutes. Fluorescence intensity was assessed using a flow cytometer (BD, USA).

### Myeloma xenograft mouse model

Mice experiment was performed under the protocol approved by the Institutional Animal Care and Use Committee of Central South University (NO. 2019sydw0105) in accordance with the Guidelines for the Care and Use of Laboratory Animals. NOD-Prkdc^scid^ Il2rg^tm1^/Bcgen mice (deficient in mature T lymphocytes, B lymphocytes and NK cells) were purchased from Jiangsu Biocytogen Co., Ltd. Mice were raised in a super pathogen-free condition. MM xenograft mouse model was established by subcutaneous injection of 1 × 10^6^ ARP-1 cells (in 100 μL of Matrigel-cell suspension mixture) into the left abdomen of mouse. Ten days later, when tumors became palpable, mice were randomized into four groups: Vehicle (control group), CHI (15.0 mg/kg), BTZ (1.0 mg/kg), and CHI (15.0 mg/kg) combined with BTZ (1.0 mg/kg). Tumor-bearing mice were treated with vehicle control, CHI (15.0 mg/kg by intraperitoneal injection (i.p), every other day for 14 days), BTZ (1.0 mg/kg i.p. 3 days weekly, interval administration). Caliper measurements of the tumor diameters were performed 3 days weekly, and the tumor volume was estimated as the volume of an ellipse using the following formula: 4/3π × (a/2) × (b/2)^2^. Tumor volume were monitored every 3 days. The animals were sacrificed at the end of the study when tumor volume reached 2000 mm^3^, at which time the tumors were excised, weighed, subjected to histological analysis and extraction of protein for Western blot analysis.

### Statistical analysis

All data are expressed as means ± standard deviation (SD) and are representative of at least three separate experiments. The two-tailed Student t-test and one-way ANOVA were used for comparisons of two or more than two groups, respectively. p < 0.05 was considered statistically significant. GraphPad Prism 7.0 software (CA, USA) was used for the analysis.

## Results

### High expression of HDAC1 is sensitive to Chidamide in MM cells

**Figure 1:**
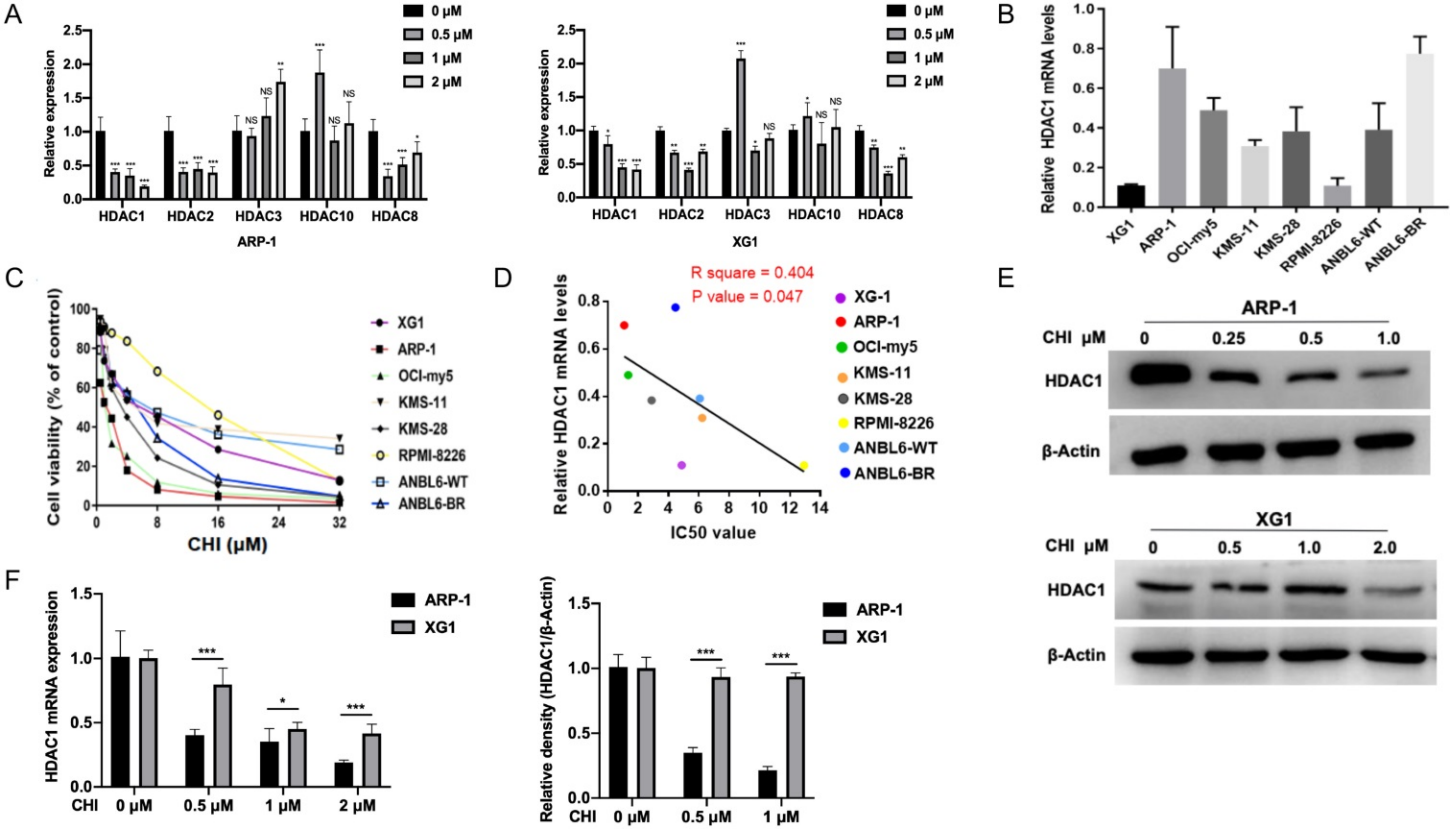
**High expression of HDAC1 is sensitive to Chidamide in MM cells**. **(A)** Relative mRNA levels of HDACs in ARP-1 and XG1 cells treated with different concentrations of CHI by qRT-PCR. **(B)** Relative mRNA levels of HDAC1 in MM cell lines by qRT-PCR. **(C)** CCK8 analysis of viability of MM cell lines treated with different concentrations of CHI. **(D)** The growth inhibition IC_50_ values in MM cell lines shows a negative correlation with relative mRNA levels of HDAC1. **(E)** The protein levels of HDAC1 were decreased with the increasing concentrations of CHI treatment for 48 hours in ARP-1 and XG1 cells. **(F)** The quantitation of mRNA and protein levels of HDAC1 in ARP-1 and XG1 cells treated with CHI. Error bars indicate mean ± SD. *p < 0.05; **p < 0.01; ***p < 0.001; NS, not significant.

As CHI was a subtype selective HDACi against HDAC1, 2, 3 and 10 11, we first investigated the mRNA levels of Class I HDACs (HDAC1, 2, 3 and 8) and HDAC10 in MM ARP-1 and XG1 cells treated with different concentrations of CHI (0.5 μmol/L, 1 μmol/L, 2 μmol/L). Among these HDACs, qRT-PCR results showed that the expression of HDAC1 was the most significant inhibition by CHI in both ARP-1 and XG1 cells (Figure 1A). Then we focused the basal mRNA level of HDAC1 in different MM cell lines. As shown in Figure 1B, among these eight MM cell lines (XG1, ARP-1, OCI-my5, KMS-11, KMS-28, RPMI-8226, ANBL6-WT, ANBL6-BR), ARP-1 showed the highest HDAC1 expression at mRNA level, while XG1 showed the lowest. ANBL6-BR was BTZ-resistant cells with a higher HDAC1 expression than ANBL-6 wild-type (ANBL6-WT) cells (Figure 1B). Yet it was noteworthy that the protein levels of HDAC1 were not completely consistent with the mRNA levels in these myeloma cell lines (Supplementary Figure S1). Subsequently, these eight MM cell lines were treated with different doses of CHI (0.5 - 32.0 μmol/L) for 48 hours. CCK-8 results showed a dose-dependent pattern of CHI cytotoxicity and the IC_50_ values of CHI were ranged from 1.1 to 12.9 μmol/L (Figure 1C). ARP-1 was the cell line that most sensitive to CHI among these eight MM cell lines, and the IC_50_ values of CHI in those MM cell lines were negatively correlated with the mRNA expression of HDAC1 (Figure 1D), suggesting that the cytotoxicity of CHI on MM cells is partly dependent on the HDAC1 expression. To confirm the effect of CHI on the expression of HDAC1 in MM cells, ARP-1 and XG1 were treated with CHI for 48 hours, Western blot showed that CHI was effective in decreasing HDAC1 in both ARP-1 and XG1 cells (Figure 1E), and moderately or faintly decreased the expression of HDAC2 or HDAC8 protein in ARP-1 and XG1 cells, respectively (Supplementary Figure S2). Furthermore, ARP-1 with the higher HDAC1 expression was more sensitive to CHI than XG1, because the mRNA and protein expression of HDAC1 in ARP-1 cells was decreased by CHI at lower dose (Figure 1F). Thus, CHI could be a promising drug for MM patients with high expression of HDAC1 in tumor cells.

### Chidamide inhibits proliferation and induces cell apoptosis and G0/G1 arrest in MM cells

**Figure 2:**
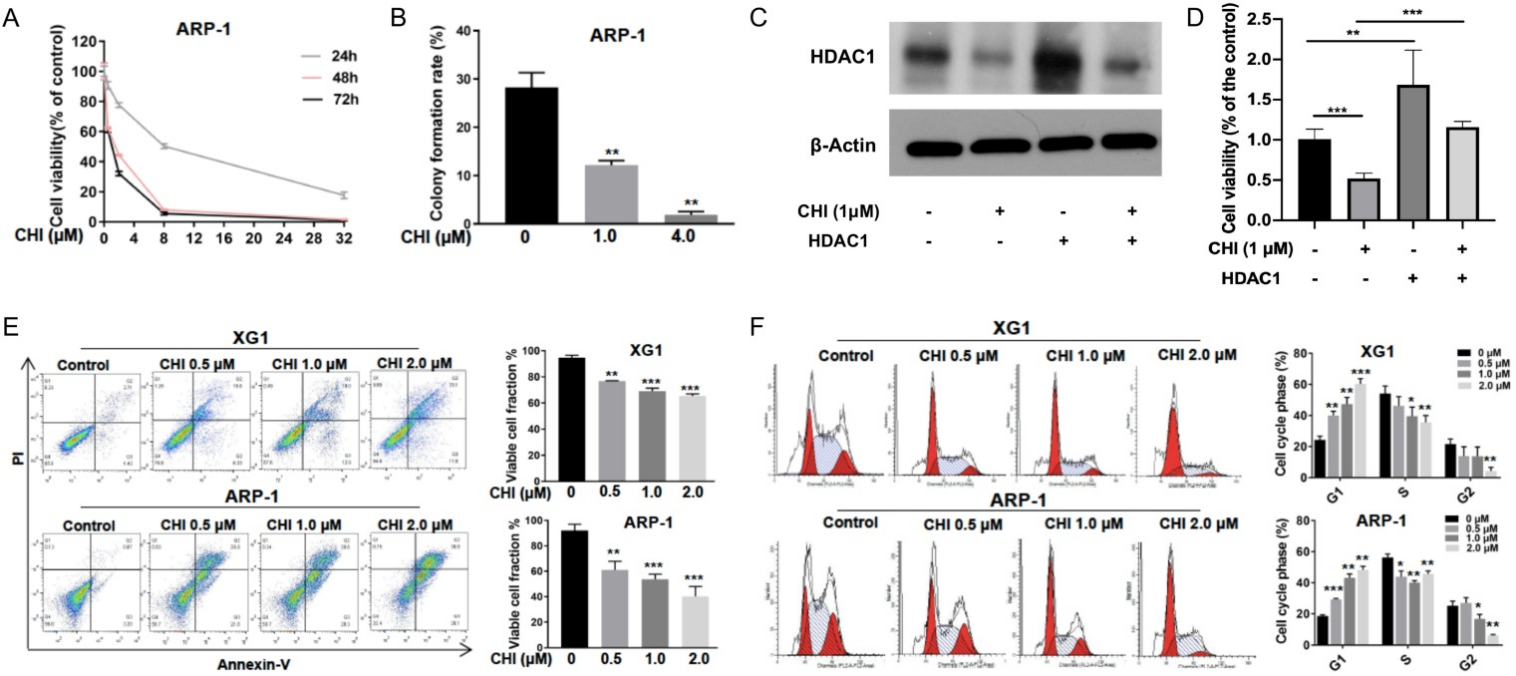
**Chidamide inhibits proliferation and induces cell apoptosis and G0/G1 arrest in MM cells**. **(A)** CCK8 analysis of viability of ARP-1 cells treated with different concentrations of CHI (0, 2.0 µmol/L, 8.0 µmol/L, 32.0 µmol/L) for 24, 48, 72 hours, respectively. **(B)** Colony forming assay showed the colony formation rate of cells treated with different concentrations of CHI (0, 1.0 μmol/L, 4.0 µmol/L) for 48 hours in ARP-1 cells. **(C)** The expression of HDAC1 in ARP-1 cells treated with 1.0 µmol/L CHI or HDAC1 cDNA or not by Western blot. **(D)** CCK-8 analysis of viability of ARP-1 cells treated with 1.0 µmol/L CHI or HDAC1 cDNA or not. **(E)** Annexin V-FITC/PI double staining analysis of XG1 and ARP-1 cells treated with different concentrations of CHI (0, 0.5 µmol/L, 1.0 µmol/L, 2.0 µmol/L) for 48 hours. Percentages of MM cell apoptosis based on three independent experiments. **(F)** Cell cycle analysis of XG1 and ARP-1 cells treated with different concentrations of CHI (0, 0.5 µmol/L, 1.0 µmol/L, 2.0 µmol/L) for 24 hours. Percentages of the subpopulation of MM cells at different cell phases based on three independent experiments. Error bars indicate mean ± SD. *p < 0.05; **p < 0.01; ***p < 0.001.

To further evaluate the effect of CHI on MM, ARP-1 cells were treated with CHI (0.5-32.0 μmol/L) for 48 hours, and then cell viabilities were examined at different time points (24, 48, or 72 hours) using CCK-8. The result demonstrated a dose-dependent and time-dependent pattern of CHI cytotoxicity in ARP-1 cells (Figure 2A). Meanwhile, the clonogenic soft agar assay showed that the colonies were dramatically decreased in ARP-1 after treatment with CHI (Figure 2B). To further validate the role of HDAC1 in the cytotoxicity of CHI on MM cells, ARP-1 cells treated with 1.0 μmol/L CHI or not were transfected with HDAC1 cDNA for 48 hours. Western blot revealed that the expression of HDAC1 protein was successfully upregulated by transfection of HDAC1 cDNA (Figure 2C), and overexpression of HDAC1 could inhibit the anti-proliferation effect caused by CHI (Figure 2D).

Subsequently, we tried to determine the effect of CHI on apoptosis and cell cycles. ARP-1 and XG1 were treated with CHI (0, 0.5, 1, 2 μmol/L), and then apoptotic cells were examined by Annexin-V staining. Dose-dependent increases of apoptotic cells were observed in both ARP-1 and XG1, moreover, more apoptotic cells were detected in ARP-1 cells compared with XG1 cells when they were treated with the same dose of CHI (Figure 2E). Cell cycles results from flow cytometry (PI staining) analysis showed that the percentage of G1 phase was significantly increased in both ARP-1 and XG1 treating with CHI, while the percentages of S phase and G2 phase were decreased (Figure 2F), indicating that CHI induced G1 arrest in MM cells. Collectively, our results showed that inhibited proliferation and induced cell apoptosis and G0/G1 arrest in MM cells.

### Chidamide increases Bortezomib effects synergistically in antagonizing MM cell growth

**Figure 3:**
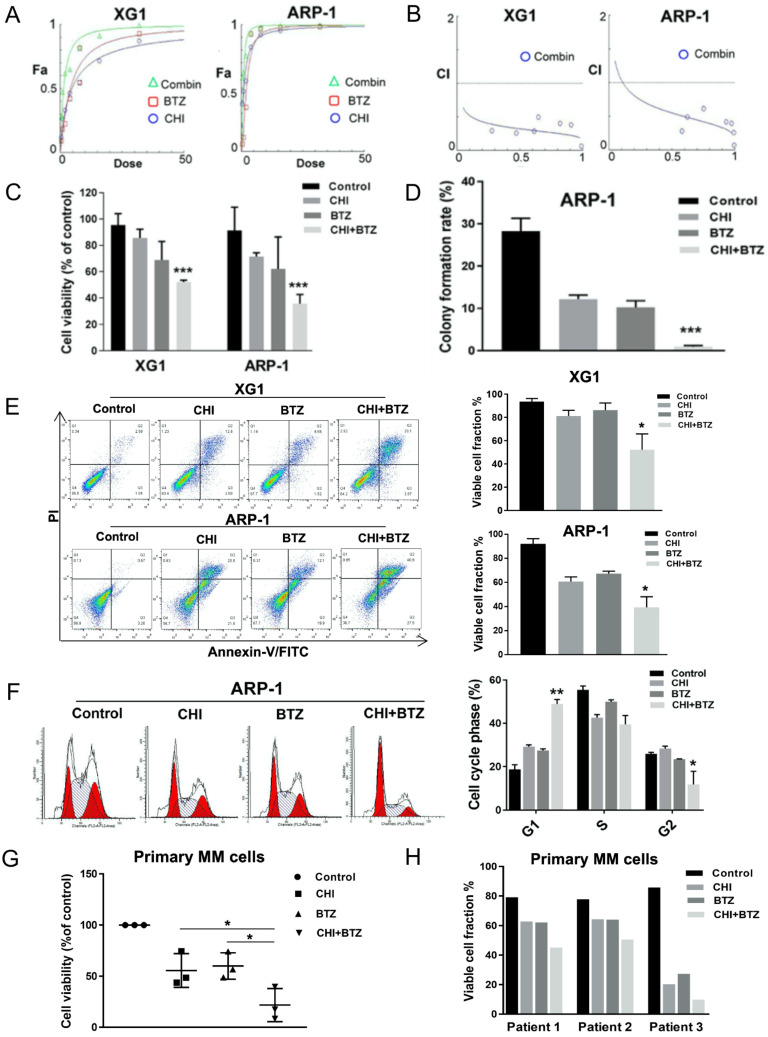
**Chidamide increases Bortezomib effects synergistically in antagonizing MM cell growth**. **(A)** The dose-effect curve of CHI and BTZ as single agent and in combinations in XG1 and ARP-1 cells using the Chou-Talalay method. **(B)** The observed CI of combination in the experiments performed and the line gives an estimation of the CI for the combination in XG1 and ARP-1 cells. **(C)** CCK8 analysis of the combination of CHI (0.5 µmol/L) and BTZ (2.0 nmol/L) in XG1 and ARP-1 cells after 48 hours of treatment. **(D)** Colony forming assay showed the colony formation rate of cells treated with CHI (1.0 µmol/L) combined with BTZ (5.0 nmol/L) for 48 hours in ARP-1 cells. **(E)** Flow cytometry (Annexin V/PI) analysis of the apoptosis of the combination of CHI (0.5 µmol/L) and BTZ (2.0 nmol/L) in the MM cell lines XG1 and ARP-1 after 48 hours of treatment. **(F)** Cell cycle analysis of ARP-1 cells treated with CHI (0.5 µmol/L) and BTZ (2.0 nmol/L) for 24 hours.** (G, H)** MM cells derived from two patients with newly diagnosed MM and a patient with relapsed/refractory MM were treated *in vitro* for 36 hours with CHI (1.0 μmol/L) and BTZ (5.0 nmol/L) in monotherapy and in combination. CCK8 and Flow cytometry (Annexin V/PI) were used to analyze the proliferation and apoptosis. Error bars indicate mean ± SD. *p < 0.05; **p < 0.01; ***p < 0.001.

Previous studies had determined that over-expression of HDAC1 promoted BTZ-resistance in MM cells 20. Our above data indicated CHI decreased HDAC1 in MM cells and its effect on MM cells was positively correlated with HDAC1 expression. We thus speculated that CHI might be synergistic with BTZ in MM cells. The synergistic effect of CHI and BTZ was investigated by using the Chou-Talalay method 22. XG1 and ARP-1 were treated with different concentrations of CHI and BTZ in monotherapy, and in combination for 48 hours, then CCK-8 assay was performed to examine cell viability and the combination index (CI) was calculated by Chou-Talalay method. As shown in Figure 3A-C, the combination of CHI and BTZ significantly accelerated cell death in both XG1 and ARP-1 cells compared with CHI or BTZ alone, moreover, both two MM cell lines displayed significant synergistic effects with CI in the synergistic range (0.3-0.7). Meanwhile, soft agar colony forming assay showed that a significant decrease in clonogenic ability was observed in ARP-1 after treatment with combination of CHI and BTZ compared with single-agent alone (Figure 3D). Above data showed that CHI induced apoptosis and G1 arrest in MM cells, we wondered whether combination of CHI and BTZ increased apoptosis and G1 arrest in MM cells. As a result, increased apoptotic cells were observed in both ARP-1 and XG1 treated with combination of CHI and BTZ than that observed in CHI or BTZ alone (Figure 3E) and increased G1 arrest was also observed in ARP-1 with the same treatment (Figure 3F). Subsequently, the synergistic effect of CHI and BTZ was explored in bone marrow monocytes derived from two newly diagnosed and one relapsed MM patients. As shown in Figure 3G and 3H, decreased cell viability and increased apoptotic cells were obtained in those cells treated with combination of CHI and BTZ compared with CHI or BTZ alone. These findings indicated that CHI was synergistic with BTZ in antagonizing MM cell growth.

### A combination of Chidamide and Bortezomib increases production of ROS dependent DNA damage and the changes of cell apoptosis and cycle pathway in MM cells

**Figure 4:**
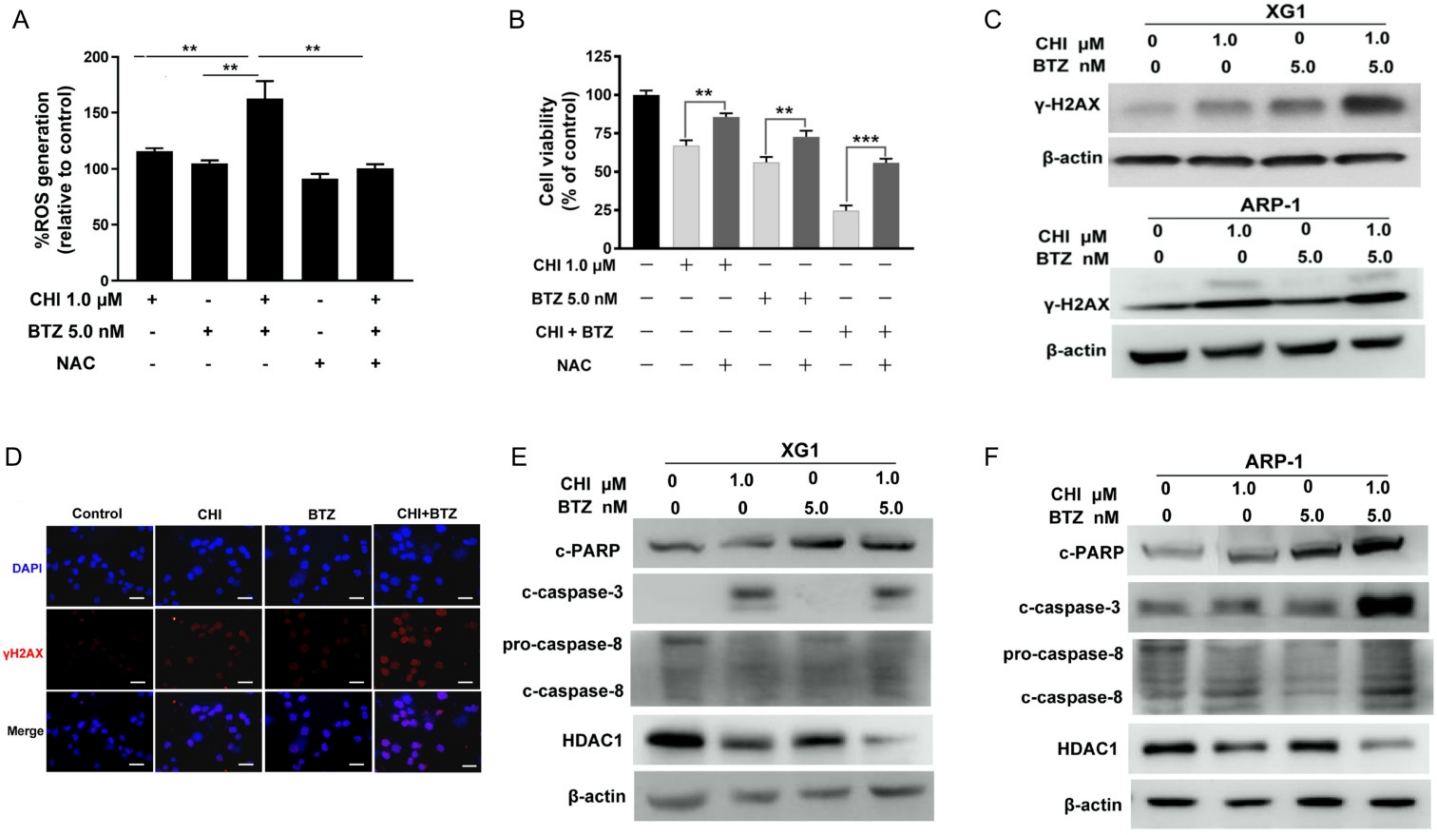
**A combination of Chidamide and Bortezomib increases production of ROS dependent DNA damage and the changes of cell apoptosis and cycle pathway in MM cells. (A)** ARP-1 cells were pretreated with or without NAC (15.0 mmol/L) for 2 hours at 37°C and then incubated with CHI (1.0 µmol/L) and/or BTZ (5.0 nmol/L) for 24 hours, then ROS generation was detected. **(B)** ARP-1 cells were pretreated with or without 15 mmol/L NAC and then treated with Chidamide or Bortezomib alone or in combination, and cell viabilities were evaluated using CCK-8 assays.** (C)** The expression of γ-H2AX in ARP-1 cells treated with CHI (1.0 µmol/L) and/or BTZ (5.0 nmol/L) were determined by Western blot. **(D)** Representative images of γ-H2AX (Red) and nuclei (Blue) in ARP-1 cells treated with single agent or combination for 24 hours by immunofluorescence assay. Scale bars represent 20 µm. **(E, F)** Western blot analysis of the expressions of cleaved caspase3, cleaved caspase8, cleaved PARP-1 and HDAC1 in XG1 (E) and ARP-1 (F) cells after 48 hours treatment with single agent or in combination. Error bars indicate mean ± SD. **p < 0.01.

Previous studies had reported that proteasome inhibitor was synergistic with HDAC inhibitor through increasing ROS 24, 25. CHI was found to induce ROS-dependent cell death in leukemia cells 11. Thus, we hypothesized that increase of ROS might be the main mechanism by which CHI synergistic with BTZ in MM cells. To confirm this hypothesis, ARP-1 was pre-treated with or without NAC, followed by treatment with CHI or in combination with BTZ. ROS assay showed that the combination of CHI and BTZ significantly increased ROS level in ARP-1 as compared with CHI or BTZ alone, moreover, combination of CHI and BTZ-induced ROS was substantially abrogated by NAC (Figure 4A). Meanwhile, the proliferative toxicities of CHI or BTZ alone and in combination on MM cells were evidently reversed by NAC (Figure 4B), suggesting that the cell death at least in part, was ROS-dependent. It was widely accepted that ROS leads to cell death mainly through inducing DNA damage. We subsequently examined the expression of γ-H2AX, a DNA damage marker, in XG1 and ARP-1 cells after treatment with CHI and BTZ alone or in combination. Western blot demonstrated that increased γ-H2AX was observed in MM cells in response to combination of CHI and BTZ compared with those cells treated with CHI or BTZ alone (Figure 4C), which was also validated by immunofluorescence staining of γ-H2AX in ARP-1 cells (Figure 4D). In addition, the expression of cleaved caspase-3, cleaved caspase-8 and cleaved PARP increased, while the expression of HDAC1 decreased in XG1 and ARP-1 cells treated with the combination of CHI and BTZ (Figure 4E, F). Thus, we thought that the synergistic anti-tumor effect of CHI and BTZ may be related with the increased production of ROS dependent DNA damage and the changes of cell apoptosis and cycle pathway in MM cells.

### Chidamide and Bortezomib synergistically inhibits MM cell growth *in vivo*

**Figure 5:**
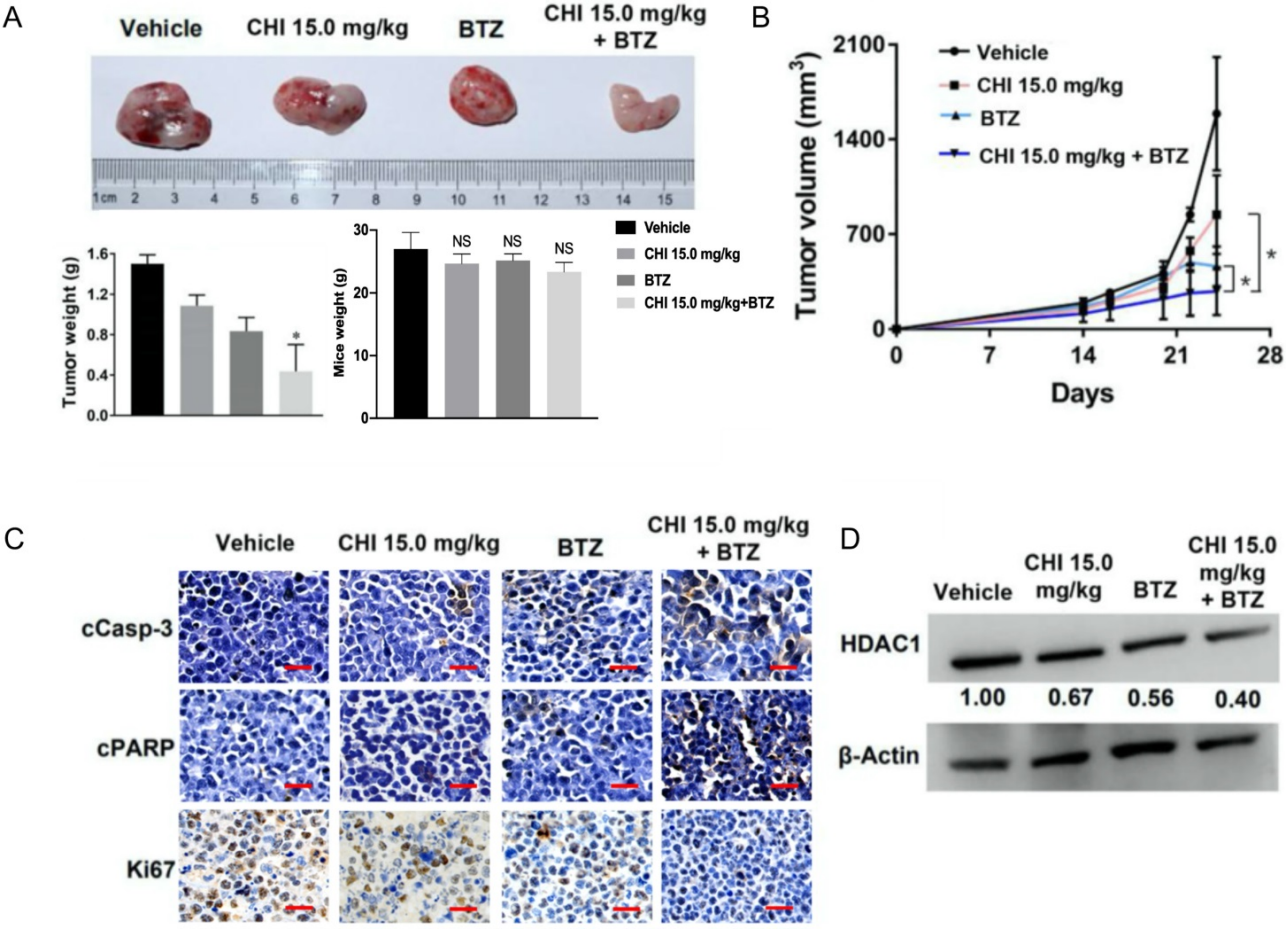
**Chidamide and Bortezomib synergistically inhibits MM cell growth *in vivo*. (A, B)** Efficacy of single-agent and combination treatment of CHI with BTZ in a human myeloma model in B-NDG mice. Tumor volumes, tumor weight and mice weight in ARP-1 cells xenografted mice model following treatment with single agent CHI and combinations with BTZ were shown (n=3). **(C)** Immunohistochemical analyses with anti-cleaved caspase-3, anti-cleaved PARP and anti-Ki-67 induced by the treatment with single agent and in combinations *in vivo*. **(D)** Western blot analysis of the expression of HDAC1 in the xenografts treated with single agent and in combinations. Error bars indicate mean ± SD. **p < 0.01; NS, not significant.

Above data determined the synergistic effect of CHI and BTZ *in vitro*, we next explored whether the synergistic effect was applied to *in vivo* MM model. Then, 1 × 10^6^ ARP-1 was injected subcutaneously into the left abdomen of B-NDG^®^ mice (NOD-Prkdc^scid^ Il2rg^tm1^/Bcgen). After 10 days, all mice were divided into four groups randomly (n=3), and then treated with CHI (15.0 mg/kg), BTZ (1.0 mg/kg), combination of CHI and BTZ, and PBS, respectively. Tumor progression was monitored by tumor volume. As shown in Figure 5A and 5B, compared to the vehicle group or single drug group, a significant decrease in tumor volume and tumor weight was observed in the combination of CHI and BTZ group and there were no significant differences in mice weight. Next, we examined the expression of proliferation markers Ki67 and apoptotic markers cleaved Caspase-3 and cleaved PARP in tumors using immuno-histochemical. Tumors from mice treated with the combination of CHI and BTZ showed a decrease in the expression of cell Ki-67 and increase of cleaved Caspase-3 and cleaved PARP compared with vehicle, CHI or BTZ alone (Figure 5C). In addition, Western blot revealed that the expression of HDAC1 was downregulated in tumors treated with the combination of CHI and BTZ (Figure 5D). These data suggested that CHI and BTZ synergistically inhibited MM cell growth *in vivo*.

## Discussion

MM is an incurable monoclonal plasma cell disorder 26. Drug-resistance induced relapse is the most important cause for the death of MM patient. Thus, it is urgent demand to develop novel drugs against MM. CHI, a novel orally HDACi has been approved by the CFDA for the treatment of PTCL 14, was found to be potential therapeutic agent in MM *in vitro* and *in vivo* 15, 17, 27. In the present study, we showed for the first time that combination of CHI and BTZ significantly increased cell death compared with CHI or BTZ alone. In addition, the underlying mechanisms by which CHI synergizes with BTZ was explored.

Multiple HDACs were over-expressed in MM cells, and played important roles in MM progression and drug-resistance 5, 20. Several HDACis especially class I HDACis have achieved excellent treatment result in MM 28-30. In this study, the effect of CHI, one of class I HDACis targeting HDAC1, 2, 3 and 10, on MM cells were investigated. Our data showed that CHI was effective in inducing cell death in MM cell lines, and the cytotoxicity of CHI on MM cell lines was positively correlated with the expression of HDAC1 in those MM cell lines. Meanwhile, overexpression of HDAC1 could reverse the anti-proliferation effect caused by CHI, which suggested that CHI affected MM cells partly dependent on HDAC1. It had been widely shown that HDAC inhibitors could induce apoptosis and cell cycle arrest, all of which eventually resulted in an inhibition of cell proliferation 11, 31. Here, our data also confirmed that CHI could induce apoptosis and G0/G1 arrest in MM cells.

BTZ, as the first proteasome inhibitor, has achieved good treatment effect in MM therapy, however, the development of BTZ-resistance attenuated its long-term utility 2, 19, 32. HDAC1 was found to be positively associated with BTZ-resistance, moreover, over-expression of HDAC1 promoted resistance to BTZ in MM cell, implicating that targeting HDACs might sensitize MM cells to BTZ 20, 33, 34. In fact, the synergistic anti-MM activities between HDACi and BTZ have been observed in several preclinical studies 24, 28. Pan-HDACi had shown clear benefit in patients with relapsed or refractory MM when combined with BTZ 35, 36. It was also showed that normal hematopoietic cells were largely unaffected when exposed to combination of BTZ and HDACi 37. These findings implicated that combination of HDACis and BTZ could be a promising therapeutic strategy for MM. In this study, we found that combination of CHI and BTZ significantly inhibited cell growth and increased apoptotic cells as compared with CHI or BTZ alone in MM cell lines as well as primary MM cells derived from 3 MM patients. Moreover, the synergistic effect of CHI and BTZ on MM was further evidenced by decreased tumor growth in MM xenograft mice treated with combination of CHI and BTZ compared with those mice treated with single drug. Taken together, our findings confirmed the synergistic effect of CHI and BTZ on MM, and provided a promising therapeutic strategy for MM.

CHI was able to induce ROS-dependent cell death in leukemia cells 11, 38 and previous studies have indicated that BTZ synergized with HDACis via increasing ROS level in MM cells 39, 40. In this study, MM cells treated with combination of CHI and BTZ showed higher level of ROS than those cells treated with single reagent. In addition, increased γ-H2AX, cleaved Caspase-3, cleaved PARP were also observed in MM cells treated with combination of CHI and BTZ compared with CHI or BTZ alone. Therefore, we concluded that CHI synergized with BTZ through increasing ROS dependent DNA damage and the changes of cell apoptosis and cycle pathway in MM cells.

In summary, our current study found that the anti-tumor effect of CHI was partly depended on the expression of HDAC1 in MM cells and CHI combined with BTZ exhibited synergistic effect in antagonizing MM cell growth *in vitro* and* in vivo*. Moreover, our data showed increase of ROS dependent DNA damage and the changes of cell apoptosis and cycle pathway were the potential mechanisms by which CHI synergized with BTZ in MM cells. In general, our findings provide a novel promising therapeutic strategy for MM patients, especially for those MM patients resistant to BTZ.

## Supplementary Material

Supplementary figures and tables.
