# Supplementary material for: Chidamide, a subtype-selective histone deacetylase inhibitor, enhances Bortezomib effects in multiple myeloma therapy
Source: J Cancer. 2021 Aug 27;12(20):6198–208. doi: 10.7150/jca.61602 (PMC8425211; doi:10.7150/jca.61602)
Supplement: Supplementary file 1 — Supplementary figures and tables. [file jcav12p6198s1.pdf]

Supplementary Figures

Supplementary Figure S1. Relative protein levels of HDAC1 in the eight MM cell lines by Western blotting.

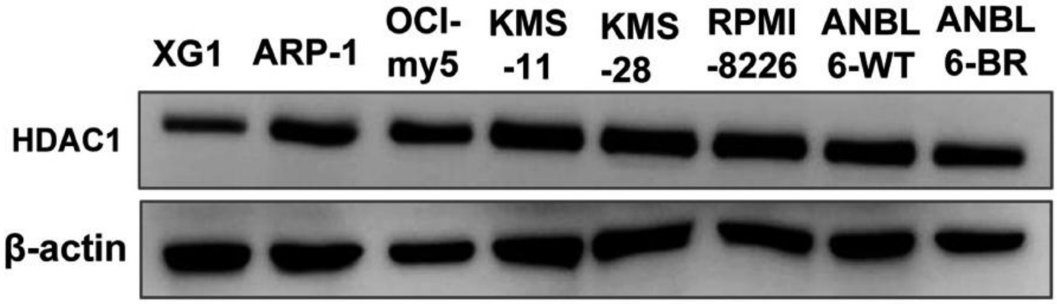

Supplementary Figure S2. The protein levels of HDAC2 and HDAC8 in ARP-1 and XG1 cells treated with different concentrations of CHI for 48 hours.

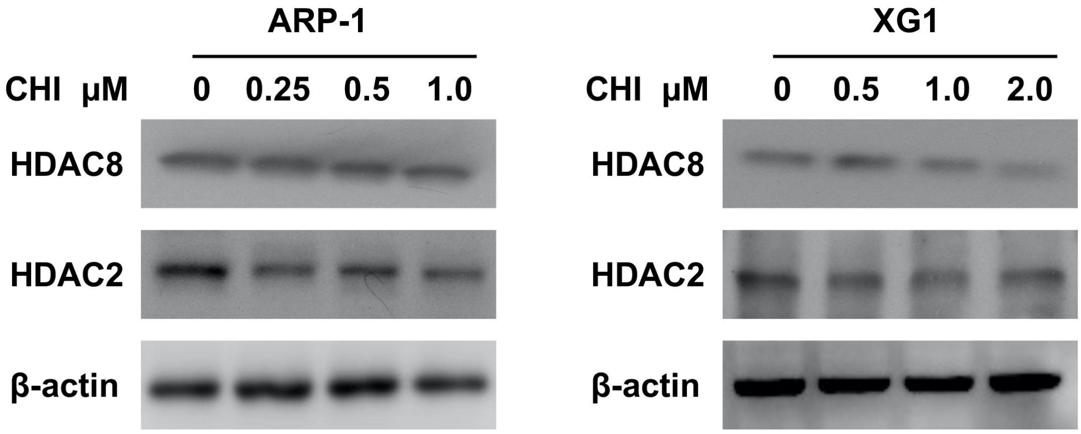

Supplementary Tables

Supplementary Table S1. Clinical data of MM patients.

	Sex	Age	classification for monoclonal protein	Clonal plasma cell percentage	FISH	DS stage	ISS stage	R-ISS stage	Previous treatment scheme
Patient 1	Male	66	IgA λ	20.5	none	ⅢA	Ⅲ	Ⅲ	Newly diagnosis
Patient 2	Female	72	IgG κ	46.5	gain (1q21); del (RB1); del (DI3S319)	ⅢA	Ⅲ	Ⅲ	3 cycles of BDT regimen
Patient 3	Female	62	κ light chain	22.4	del (RB1); del (DI3S319)	ⅡA	Ⅱ	Ⅱ	Newly diagnosis

Supplementary Table S2. The sequences of primers.

Gene	Primer sequences (5’-3’)
HDAC1-F	GGTCCAAATGCAGGCGATTCCT
HDAC1-R	TCGGAGAACTCTTCCTCACAGG
HDAC2-F	AAAGTCTGCTACTACTACGACG
HDAC2-R	TTATGGGTCATGCGGATTCTAT
HDAC3-F	TTCAATATCCCTCTACTCGTGC
HDAC3-R	AGGTTTTCAAAGATTGTCTGGC
HDAC8-F	TCGCTGGTCCCGGTTTATATC
HDAC8-R	TACTGGCCCGTTTGGGGAT
HDAC10-F	CAGTTCGACGCCATCTACTTC
HDAC10-R	CAAGCCCATTTTGCACAGCTC
β-actin-F	GTCTTCCCCTCCATCGTG
β-actin-R	TTCTCCATGTCGTCCCAG
